# COVID-19: hypofractionation in the Radiation Oncology Department during the ‘state of alarm’: first 100 patients in a private hospital in Spain

**DOI:** 10.3332/ecancer.2020.1052

**Published:** 2020-05-28

**Authors:** Luis Larrea, Enrique López, Paola Antonini, Verónica González, Miguel Ángel Berenguer, Maria Carmen Baños, Jose Bea, Jose Domingo

**Affiliations:** 1 Radiation Oncology Department, Hospital Vithas Valencia Consuelo, 46007, Spain; 2 Radiophysics Department, Hospital Vithas Valencia Consuelo, 46007, Spain

**Keywords:** COVID-19, hypofractionation, Fractionation Index (FI)

## Abstract

During the COVID-19 pandemic, Spain declared a ‘state of alarm’ on 14 March 2020. In our Radiation Oncology Department, experienced in administering hypofractionated treatments (partial irradiation in breast cancer, moderate hypofractionation in localized prostate cancer, etc), we have increased the hypofractionated treatment indications. We are only deferring the start of non-urgent treatments such as prostate tumours under androgen deprivation or benign brain tumours which are candidates for radiosurgery such as meningiomas or acoustic neuroma.

In this hypofractionation era we find that we have decreased the number of sessions per patient and that we can evaluate the last years with the fractionation index (FI) (calculated by dividing the total number of fractions administered in the department by the total number of patients treated). We have gone from 14.4 in 2018 to 13.78 in 2019, excluding brachytherapy.

We report the results of the first 100 patients who have experienced radiotherapy treatment since the state of alarm (66 women and 34 men). In these patients, the FI is 12.12—lower than previous years.

## Introduction

In Spain, on 14 March 2020, a ‘state of alarm’ was declared due to the COVID-19 pandemic, forcing citizens to be confined at home with movement restrictions. Previously, at the Radiation Oncology Department, we established major indication of hypofractionated treatments in the majority of new patients attended, according to the recommendations of national and international societies.

On the other hand, in those patients where it was possible, we delayed some treatments until the end of the maximum contagion period, for example, prostate cancer under androgen deprivation (AD) therapy or with low-grade staging, as well as radiosurgery for benign brain tumours, such as meningioma or acoustic neuroma.

Our department has two linear accelerators equipped with Image-Guided-Radiation-Therapy (*ConeBeamCT*) and the ability to perform Intensity-Modulated-Radiation-Therapy (IMRT) and Volumetric-Arc-Therapy (VMAT). We also have brachytherapy equipment for high dose rate and low dose rate. Our experience is to treat an average of 800–1200 patients per year.

## Materials and methods

Between 16 March and 16 April 2020, we analysed the first 100 consecutive patients who began treatment in our department after the declaration of the ‘State of Alarm’. We analysed the number of fractions or sessions per patient and treatment during this period. This was defined as ‘Fractionation Index’ (FI). It is calculated by dividing the total number of sessions by the total number of patients registered in our management information system (*Mosaiq***®-**Elekta®). We included all patients treated with external beam radiation therapy, excluding brachytherapy procedures.

Previously, as we were indicating more hypofractionated treatments in this Radiation Oncology Department, we calculate the FI for 2018 and 2019. We reported in 2018, with 1,011 patients a fractionation index of 14.48 sessions per patient, number that decreased in 2019 to 13.78 sessions with 854 treated patients.

The main reason for indicating hypofractionated treatments during the COVID-19 pandemic is to minimise exposure and risk of contagion of patients without reducing the effectiveness of the treatments. Our attitude was to establish a better way to treat all patients who can benefit from radiotherapy; not to delay the onset of any patient whose deferral may worsen the prognosis of their disease; try to help the patient attend treatment as few times as possible and not increasing toxicity and maintaining the possibility of the best result. The hypofractionated treatments we indicated during the time of COVID-19 are supported mainly by international guidelines and phase III clinical trials [1–19].

To preserve the quality criteria of treatments, the tolerance limits protocols, quantitative analyses of normal tissue effects in the clinic and Radiation Therapy Oncology Group were adapted to the new hypofractioned schemes, estimating the biological equivalent dose (BED).

## Results

We report the first 100 patients (66 women and 34 men). The mean age in women was 58-year old and it was 66-year old in men.

The number of patients included in each subgroup, treatment intention and tumours location are detailed in Table 1.

In summary, treatment indication has been: 55 adjuvant treatments, 18 radicals, 19 oligometastatic, 4 recurrences, 3 palliative and 1 neoadjuvant.

**Figure 1.**
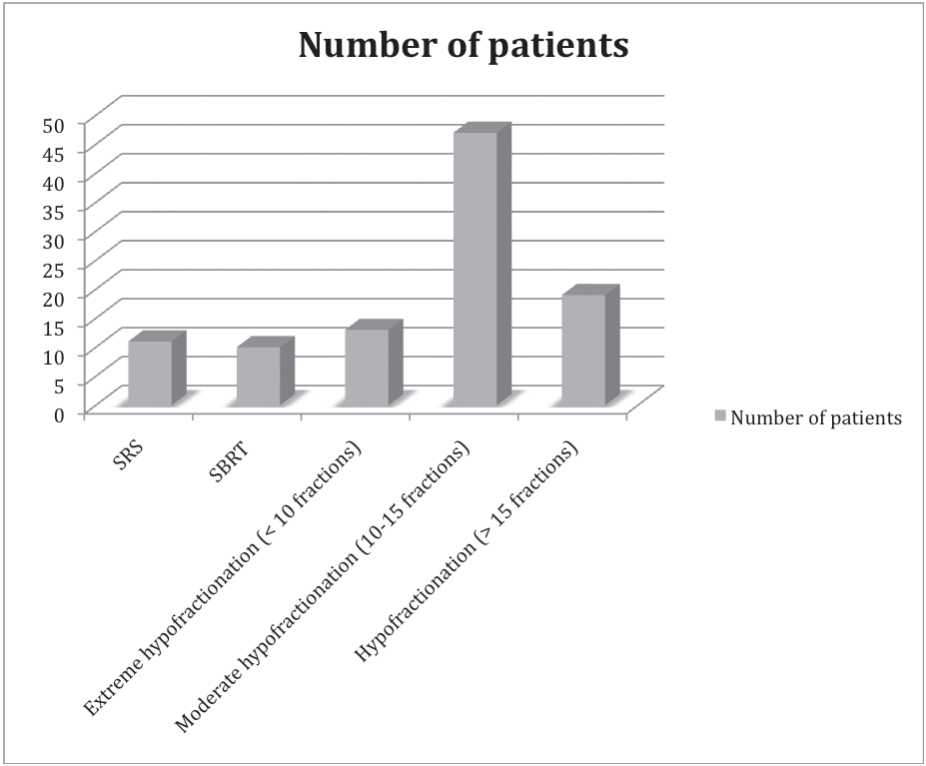
Number of patientes represented according number of sessions and technique.

Treatment by techniques: 69 patients had 3D-IMRT, 11 radiosurgery, 10 VMAT, 10 SBRT (Figure 1).

The first 100 patients treated that we evaluated shows that the FI is 12.12 sessions per patient, this is a very low average compared to standard fractionation. We consider that these are safe and recognised treatments, recommended in oncology guidelines.

During this time we delayed nine patients, seven patients with prostate cancer in whom indicated external beam radiotherapy has been deferred and two patients with acoustic neuroma. Of these 7 prostate cancer patients, 5 of them we will indicate 20 sessions (2 of them are under AD and 3 low grade) and the other 2 patients will be treated with combined modality (12 sessions and high-dose-rate brachytherapy boost, both are under AD).

If we include these 7 prostate cancer patients, the FI would be 12.48 fractions per patient, but maybe we will treat some of them with SBRT technique with 5–8 fractions. Adding also 2 SRS, this FI will be lower.

## Conclusion

The current average FI is much lower than the average standard treatments.

This change in our prescription using hypofractionated schemes allowed us to treat patients in a more isolated way so they have fewer contacts in the waiting room. Furthermore, it allows us to disinfect the equipment between patients. We had not report COVID-19 among our staff or patients.

In the near future, we also will receive patients who are now suffering delays in diagnosis and surgery, then we will gradually adapt the treatments for them.

Due to the low FI, hypofractionated radiotherapy treatments can be more cost-effective during the COVID-19 pandemic.

FI can be an easy and effective formula to evaluate a radiation therapy department.

## Conflicts of interest statement

Dr Larrea is a member of the *e*cancer editorial board.

## Funding declaration

No funding to declare.

## Figures and Tables

**Table 1.** Treatments performed by intention, techniques and number of sessions.

Tumour location	Treatment intention (*N*)	Type of treatment (*N*)	Fractions per Treatment	Total number of fractions	Number of patients
Breast	Adjuvant	APBI (12) RTC-3D-IMRT (40)	5 15	660	52
Prostate	Radical (7) Biochemical relapse (2)	VMAT	20 25	140 50	9
Brain metastases (oligometastases)	Radical	SRS	1	8	8
Lung primary	Palliative (3) Radical (6) Adjuvant (1)	RTC-3D (3) IMRT (6) IMRT (1)	15-20 20 20	51 120 20	10
Lung metastases	Radical (3)	SBRT	1-3	7	3
Glioblastoma	Relapse (2)	SRS FSRS	1 4	5	2
Larynx	Radical (2)	IMRT	16	32	2
Adrenal metastases (oligometastases)	Radical (2)	SBRT	3	6	2
Skin	Adjuvant	RTC-3D	23	23	1
Pancreas	Neoadjuvant	IMRT	15	15	1
Brain hemangiopericytoma	Radical	FSRS	5	5	1
Parotid gland	Adjuvant	IMRT	25	25	1
Anal canal	Radical	IMRT	28	28	1
Supraclavicular tumour	Radical (oligometastases)	SBRT	5	5	1
Vaginal recurrence	Radical (oligometastases)	IMRT	12	12	1
Total				1212	100

RTC-3D, Tridimensional Conformal Radiotherapy; APBI, Accelerated Partial Breast Irradiation; IMRT, Intensity-Modulated-Radiation-Therapy; VMAT, Volumetric-Arc-Therapy; SRS, Stereotactic radiosurgery; SBRT, Stereotactic Body Radiation Therapy.
